# Mitigating aflatoxin B1 in high-moisture sorghum silage: *Aspergillus flavus* growth and aflatoxin B1 prediction

**DOI:** 10.3389/fmicb.2024.1360343

**Published:** 2024-05-23

**Authors:** Mariana Gonda, Caterina Rufo, Jose L. Gonzalez-Andujar, Silvana Vero

**Affiliations:** ^1^Laboratorio de Biotecnología, Área Microbiología, Departamento de Biociencias, Facultad de Química, Universidad de la República, Montevideo, Uruguay; ^2^Laboratorio de Alimentos y Nutrición, Instituto Polo Tecnológico, Facultad de Química, Universidad de la República, Pando, Uruguay; ^3^Instituto de Agricultura Sostenible (CSIC), Córdoba, Spain

**Keywords:** *Aspergillus flavus*, aflatoxin B1, sorghum silage, initial aerobic phase, fungal spoliage

## Abstract

*Aspergillus flavus* (*A. flavus*), a frequent contaminant in silage, is a significant producer of aflatoxins, notably the potent carcinogen aflatoxin B1. This contaminant poses a potential risk during the initial aerobic phase of ensiling. The present work studied the impact of temperature on *A. flavus* growth and aflatoxin B1 production in laboratory-scale sorghum silos during the initial aerobic phase. Growth curves of *A. flavus* were generated at various temperatures and modeled with the Gompertz model. Results indicated that the optimal temperature range for the maximum growth rate in sorghum mini-silos is between 25 and 30°C. Mold biomass and aflatoxin B1 levels were quantified using qPCR and HPLC, respectively. A predictive model for aflatoxin B1 synthesis in the initial ensiling phase was established, in function of grain moisture, external temperature, and time. Within the studied range, *A. flavus’s* initial concentration did not significantly influence aflatoxin B1 production. According to the model maximum aflatoxin production is expected at 30% moisture and 25°C temperature, after 6 days in the aerobic phase. Aflatoxin B1 production in such conditions was corroborated experimentally. Growth curves and aflatoxin B1 production highlighted that at 48 h of incubation under optimal conditions, aflatoxin B1 concentrations in mini-silos exceeded national legislation limits, reaching values close to 100 ppb. These results underscore the risk associated with *A. flavus* presence in ensiling material, emphasizing the importance of controlling its development in sorghum silos.

## 1 Introduction

Intensive animal production systems based on pastoral schemes require energy supplementation to achieve high production levels and great efficiency (Bartaburu, 2003). The combination of high water and protein content with low fiber in winter pastures creates an imbalance at the ruminal level due to the lack of energy to utilize that protein (Scarpitta, 2008). Supplementation with high-moisture grain silage could be a good strategy to efficiently utilize winter pasture. High-moisture grain silage is produced using grain harvested with a moisture content between 23 and 40%, with the optimum level being between 28 and 32%, and is preserved without prior drying under anaerobic conditions (Scarpitta, 2008; Rovira and Velazco, 2012). Silage production process can be divided into four well-differentiated phases (Pahlow et al., 2003): an initial aerobic phase that occurs immediately after harvesting until the oxygen is depleted; a second phase where fermentation occurs; a third stable storage phase and, finally, a feed-out phase when the feeding face of the silo is opened and the material is exposed to air.

In the presence of oxygen, aerobic microorganisms, such as fungi, can grow, and consume the ensiled material leading to depletion of nutrients and dry matter. Fungal growth may also reduce the palatability of ensiled material and pose a potential risk to animal health, as certain spoilage fungi can produce mycotoxins (Alonso et al., 2013).

Proper ensilage practices aim to minimize the contact of ensiled material with oxygen, though it temporarily occurs during the initial aerobic phase, which should not exceed 1–3 days (Piltz and Kaiser, 2004), and in the feed-out phase. In addition, aerobic deterioration can also be detected due to poor silo performance or damage caused by rodents, birds, or weather factors such as hail. Therefore, fungal growth and the emergence of associated problems are potential risks throughout the entire process.

Silage contamination is mainly associated with various species within *Aspergillus, Fusarium,* and *Penicillium* genera (Cheli et al., 2013; Wambacq et al., 2016). The predominance of one over the other depends on numerous factors, including the characteristics and quality of the ensiled material, the environmental conditions during storage, and the methods employed in the silage process. Different authors (Keller et al., 2012; del Palacio et al., 2016; García Y Santos et al., 2022) found that *A. flavus* was the main contaminant species in sorghum silages.

*Aspergillus flavus* is one of the major producers of aflatoxins, which are toxic, mutagenic, and carcinogenic secondary metabolites. The four majors naturally produced are aflatoxin B1, G1, and their dihydroxyderivates B2 and G2, respectively. Aflatoxins are classified by the International Agency for Research on Cancer (IARC) as Group 1, which means they are carcinogenic to humans (IARC, 2002). Within this group, aflatoxin B1 is considered the primary toxin and the most potent natural carcinogen (Ogunade et al., 2018).

The issue that arises when detecting *A. flavus* as a common contaminant in high-moisture sorghum grain silage is the potential risk of the presence of aflatoxins, particularly aflatoxin B1. Notably, once aflatoxins are synthesized within the silage, their complete eradication is unattainable.

Consuming food contaminated with aflatoxins (B1, B2, G1, and G2) is associated with toxicity, carcinogenicity, and reduction in animal productivity. Feeding dairy cattle with feedstuffs contaminated by aflatoxin B1 poses a problem for the dairy industry, as this metabolite undergoes biotransformation in animals, giving rise to aflatoxin M1, which is excreted in milk (Sassahara et al., 2005). Aflatoxin M1 is resistant to thermal and pasteurization processes; therefore, it could potentially affect humans through the consumption of contaminated milk, as it is considered as possibly carcinogenic to humans (IARC, 2002; Marchese et al., 2018).

Considering that *A. flavus* grows in an oxygen-rich environment and that the initial aerobic phase of silage could last 72 h, a thorough examination of how this mold thrives within silage and its concomitant production of aflatoxin B1 is required. Furthermore, understanding how different external factors impact mold growth and aflatoxin production would allow us to predict and prevent the growth of *A. flavus* at the beginning of this process and, consequently, inhibit aflatoxin B1 contamination.

In this context, the objective of the present work was to analyze the effect of temperature on *A. flavus* growth and aflatoxin B1 production on laboratory-scale sorghum silos (mini-silos), in the initial aerobic phase. In addition, it aims to develop a predictive model for aflatoxin B1 synthesis, in the initial phase of ensiling, considering external environmental factors, *A. flavus* initial concentration, and time.

## 2 Materials and methods

### 2.1 Pathogen

A native strain of *A. flavus* (PJA) isolated from poultry feed, belonging to the culture collection from Área Microbiología, Facultad de Química, UdelaR (Montevideo, Uruguay) was used in this study. The culture was maintained on Potato Dextrose Agar (PDA) at 4°C.

### 2.2 Mini-silos

Sorghum mini-silos with air leakage were prepared following the method of Gonda et al. (2019). High tannin sorghum grains (Sorghum bicolor) were ground in a blender (Philips Hr2109) at high speed for 30 s, sterilized by autoclaving (121°C, 15 min), and then dried at 60°C to constant weight. The dried sorghum was moistened with sterile distilled water to reach the desired humidity level and inoculated with 20 μL/g of a conidial suspension of *A. flavus* PJA, resulting in a predetermined concentration of conidia per gram of sorghum. Conidial suspensions were prepared by collecting conidia from 4-day-old colonies grown in PDA at 28°C, in a 0.9% saline solution with 0.1% Tween 80. The conidial concentration was determined using a hemocytometer. Mini-silos were prepared by compacting the inoculated mixture into 15 mL sterile centrifuge tubes. A sterile 0.45 μm membrane filter was inserted into the cap of each tube to simulate air leakage.

### 2.3 *Aspergillus flavus* biomass quantification

After incubation, the content of each mini-silo was freeze-dried and homogenized. Samples of 3 g were placed in a sterile bag containing 30 mL of sterile water and the mixture was homogenized in a laboratory blender (Stomacher 400, Seward, United Kingdom) for 1 min. Total DNA was extracted from all the homogenates. Two milliliters of each suspension were centrifuged for 1 min at 10.000 rpm. Pellets were reconstituted in 750 μL of a lysis solution and total DNA was extracted using the ZR Fungal/Bacterial DNA MiniPrep kit (ZymoResearch). Aliquots of DNA solutions obtained from the samples were used to determine the concentration of *A. flavus* by qPCR, as described by Gonda et al. (2019). All determinations were performed in duplicate.

### 2.4 *Aspergillus flavus* growth curves in ensiled sorghum grain

Growth curves of *A. flavus* in ensiled sorghum grain with air leakage and a humidity level of 30% (0.36 mL of sterile distilled water/g sorghum) were established at different temperatures (15, 20, 25, and 30°C) (Table 1). For each temperature, six mini-silos were prepared in duplicate, following the previously described method. The mini-silos were then retrieved and processed to measure the quantity of *A. flavus* DNA per gram of sorghum by qPCR as described above. A logarithmic transformation was applied to *A. flavus* DNA concentration values to achieve a normal distribution of the data, which was tested using a Shapiro–Wilk test.

**Table 1:** Models and their equations.

Model	Equation
Gompertz	$Y=A*\exp\left(-\exp\;\left\{\;\left[\;\mu \max*\left(e^{1}\right)*\left(\lambda -t\right)\right]/A+1\right\}\right)$
Logistic	$Y=A/\left(1+\exp\;\left\{\frac{\left[4*\mu \;\max*\left(\lambda -t\right)\right]}{A}+2\right\}\right)$

The Gomperz and Logistic models (Arroyo et al., 2005) were used to fit the growth curves, as they allow for estimating biological parameters. Nonlinear regression analysis was performed to fit the growth curves to data using the “nls” function (Nonlinear Least Squares) from the nlsMicrobio package in the R software. The initial values for the model parameters were obtained from Infostat software.

The goodness of fit was evaluated using the pseudo-*R*^2^ (Kvålseth, 1985) and the Root Mean Square Error (RMSE), which measures the residual variability between predicted and observed data (Egea-Cobrero et al., 2020). A scale of RMSE meaning is: <5; excellent prediction; 5–10; very good prediction; 10–15; good prediction; and > 15, insufficient prediction (Hemmert et al., 2016).

The corrected Akaike information criterion (AICc) was calculated to select the most accurate model (Burnham and Anderson, 2002). Models with AICc values that differ by less than 2 units do not present differences in terms of their plausibility.

The analysis was performed using the “qpcR” and “Metrics” packages from R software, while the graphical representations were created using the “plotfit” function in the “nlsMicrobio” package (RStudio Team, 2021).

### 2.5 Variables affecting aflatoxin production

Response surface methodology (RSM) based on central composite design (CCD) was used to determine the simultaneous effect of different variables on aflatoxin B1 production by *A. flavus* in ensiled sorghum grain in the presence of air. Design-Expert, Version 13 (STAT-EASE Inc., Minneapolis, United States), was used to perform the experimental design and statistical analysis. Four variables, including silage humidity, initial contamination with *A. flavus*, incubation temperature, and time, were included in the design at five levels. Twenty-seven experiments were conducted, each involving a mini-silo prepared, inoculated, and incubated under specific conditions. These experiments included three replicates at the central point. Aflatoxin B1 production was used as the response variable. An empirical model was obtained to relate the response with the experiment’s variables. Using this model, the optimal values of significant variables were determined to maximize aflatoxin B1 production. The model was experimentally validated in duplicate, and the theoretical maximum concentration of aflatoxin B1 production predicted by the model was compared with the experimental value obtained under those conditions.

Sorghum mini-silos were prepared following the previously described method, at different moisture levels and with different initial contamination of *A. flavus*. Different volumes of sterile distilled water (0.10, 0.20, 0.36, 0.60, and 0.80 mL/g) were added to dried sorghum to obtain moisture levels of 13.2, 20, 30, 40, and 46.85%, respectively. In all cases, moisture levels were determined by drying the samples at 60°C in triplicate until a constant weight was achieved. Preliminary experiments were conducted to determine the precise quantity of water required to achieve the targeted moisture level for the experiment, consistently measuring moisture based on dry weight.

Conidial suspensions of *A. flavus* at different concentrations were prepared as described above to achieve initial contamination levels of 10^2.32^ conidia/g, 10^3^ conidia/g, 10^4^ conidia/g, 10^5^ conidia/g, 10^5.68^ conidia/g of ensiled sorghum. Once prepared, mini-silos were incubated for 2, 4, 6, 8, or 9 days at 3, 10, 20, 30, or 37°C as corresponded to each trial.

After incubation, mini-silos were analyzed for aflatoxin B1 production. The content of each mini-silo was placed in a 50 mL sterile centrifuge tube and weighed for water content determinations. Samples were stored at −20°C for 24 h before lyophilization. After the samples were completely dried, they were weighed and ground to obtain a homogeneous sample.

### 2.6 Aflatoxins extraction and quantification

Aflatoxin B1 concentration was determined by high-performance liquid chromatography (HPLC) with fluorescence detection and post-column derivatization according to the AOAC Official Method 990.33 with some modifications.

Briefly, 5 g of each dried mini-silo was placed in a sterile bag containing 25 mL of a 70% methanol solution and the mixture was homogenized in a laboratory blender for 3 min (Stomacher 400, Seward, United Kingdom). Homogenates were filtered through a Whatman No. 1 paper and 10 mL of the filtered were collected and transferred into a 150 mL separatory funnel. For the partition, 10 mL of hexane was added and shaken gently for 30 s. After the layers got separated, the hexane layer was discarded. Then, aflatoxins were extracted from the aqueous phase twice with 5 mL of dichloromethane. The combined dichloromethane extracts were dried with Na_2_SO_4_, evaporated to dryness under reduced pressure (Rotavapor®R-114, Buchi, Switzerland) and resuspended in 0.2 mL of Acetonitrile HPLC grade (Merck, Alemania). For matrix analysis, 5 g of dry ground high tannin sorghum grains were extracted with the method described above.

Previous to HPLC analysis precolumn derivatization of the samples with trifluoroacetic acid (TFA) was carried out according to the AOAC Official Method 994.08 with some modifications. Derivatization solution was prepared by mixing 10 mL of trifluoroacetic acid with 5 mL of glacial acetic acid and 35 mL of distilled water. 50 μL of the sample was mixed with 117 μL of derivatization solution and then heated for 9 min in a 65°C water bath. When the mixture was at room temperature, 20 μL was injected in the HPLC in a system with a pump Waters 510 (Waters, Millipore Corporation), an injection valve equipped with a 20-μL loop, a C-18 column (150 mm × 4.60 mm, Phenomenex) and a Shimadzu Fluorescence detector RF-10A XL. Data were processed using Peak Simple version 4.26 software. The analysis was performed at room temperature with a flow rate of 1 mL/min using isocratic elution with water, methanol, and acetonitrile (70:15:25, v/v/v) as mobile phase. Fluorescence detection was performed at excitation and emission wavelengths of 360 and 440, respectively.

A calibration curve was generated by duplicate analysis of five different concentrations of Aflatoxin HPLC standard (2.0 μg/mL B1, G1 and 0.5 μg/mL B2, G2 in Acetonitrile, Trilogy, USA).

The recovery rate was determined by duplicate spiking 100 μL of Aflatoxin HPLC standard (2.0 μg/mL B1, G1, and 0.5 μg/mL B2, G2 in Acetonitrile, Trilogy, United States) into 14 mL of a 70% methanol extract obtained from 5 g of dry ground high tannin sorghum grains resuspended in 25 mL of 70% methanol and extracted as previously described.

## 3 Results

### 3.1 Modeling of growth curves

Both Gompertz and Logistic models fitted very well to the data set. In all cases, Pseudo-*R*^2^ was above 0.97 and RMSE showed excellent predictions (Table 2). The values obtained for the Gompertz model in growth curves at 20 and 30°C were lower than those of the Logistic models, indicating that the Gompertz equation describes mold growth better. However, this trend was not observed for growth at 15°C, where the Logistic model was found to be more plausible with a lower AICc. No significant difference between the two models was observed for *A. flavus* growth at 25°C, as the AICc difference was less than 2 units. Consequently, the Gompertz model was selected as the most plausible model to represent *A. flavus* growth at different temperatures.

**Table 2:** The goodness of fit criteria for Gompertz and Logistic growth models at different temperatures.

	Gompertz model			Logistic model		
Growth temperature	RMSE	Pseudo *R*^2^	AICc	RMSE	Pseudo *R*^2^	AICc
15°C	0.131	0.976	−3.79	0.114	0.982	−7.016
20°C	0.11	0.990	−8.37	0.12	0.99	−5.74
25°C	0.19	0.970	0.063	0.19	0.97	−0.92
30°C	0.079	0.996	−15.73	0.099	0.994	−10.42

RMSE, Root mean square error; AICc, Corrected Akaike information criterion.

**Figure 1:**
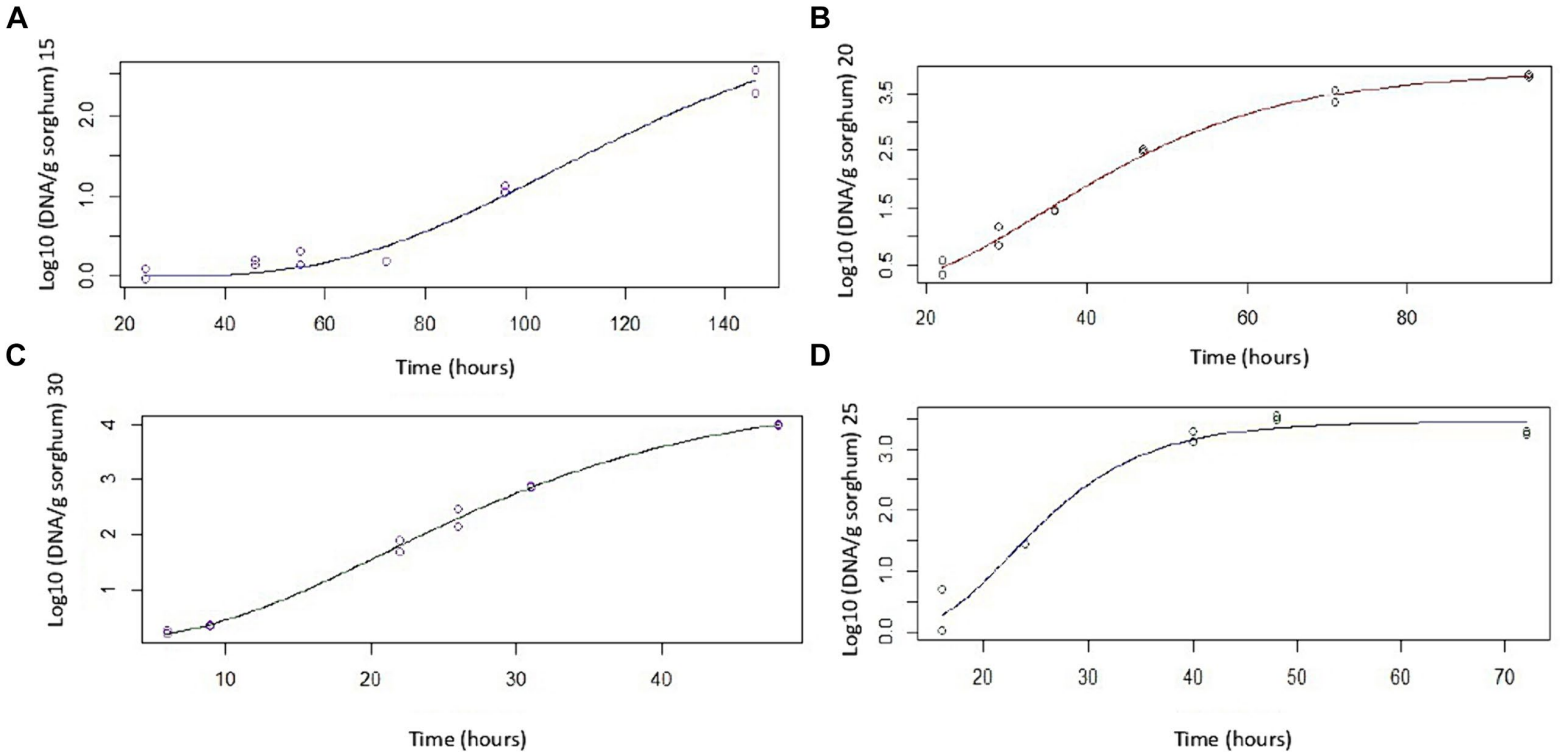
*Aspergillus flavus* growth curves fitted with Gompertz model at different temperatures: **(A)** Growth at 15°C, **(B)** Growth at 20°C, **(C)** Growth at 30°C, and **(D)** Growth at 25°C.

Figures 1A–D depict *A. flavus* growth at 15, 20, 25, and 30°C, respectively. The estimated values of Gompertz model parameters at different temperatures are shown in Supplementary Table S1. The maximum growth rate (μ max) for each temperature was obtained from the corresponding equation. The growth rates of *A. flavus* in sorghum mini-silos rapidly increased up to 25°C. At 30°C, a slightly lower value was obtained, although it was not significantly different from the value obtained at 25°C, calculated by LSD Fisher Test *α* = 0.05 (data not shown). The maximum growth rate of *A. flavus* in mini-silos should be between 25 and 30°C (Supplementary Table S1).

### 3.2 Aflatoxin B1 quantification

The standard curve generated for quantification by HPLC revealed a strong linear relationship (*R*^2^ = 0.996) between the fluorescence intensity and aflatoxin B1 concentration (ng/g). Linearity was observed over the range between 4 and 200 ng/mL of aflatoxin B1. The detection limit was (0.78 ± 0.05) ng/g of aflatoxin B1 on sorghum and the recovery rate was 85% ± 14%. High tannin sorghum grain extract did not show components with a retention time that overlapped the peak corresponding to aflatoxin B1, which presented a retention time of 10.9 min.

### 3.3 Determination of the factors that influence aflatoxin B1 production

Response surface methodology using CCD was applied to determine the influence of different variables on aflatoxin B1 production (Supplementary Table S2). The results were analyzed through ANOVA and a model involving significant factors was generated (Table 3). In the assayed range, linear terms of temperature and quadratic terms of temperature, moisture, and time were significant for the responses (*p* < 0.05) but *A. flavus* initial concentration was not significant (*p* > 0.05). Despite the results, linear terms of moisture and time were included in the equations to generate a hierarchical model. The fitness of the model was checked with *R*^2^ and the statistical significance was assessed by ANOVA (*p* = 0.0001). The CCD was fitted with the following second equation model for aflatoxin B1 production: LOG10 (B1 aflatoxin) = −0.013*moisture^2^−9.58 ×10^−3^*temperature^2^-0.11*time^2^ + 0.74*moisture +0.47*temperature + 1.51*time − 18.31, with an *R*^2^ = 0.84.

**Table 3:** ANOVA of the central composite design showing the effect of factors on B1 aflatoxin production.

Source	Sum of squares	Degrees of freedom	Mean square	*F* value	*p* value prob. > *F*
Model	54.27	6	9.04	17.48	<0.0001
A-Moisture	0.70	1	0.70	1.34	0.2599
B-Temperature	17.81	1	17.81	34.44	<0.0001
C-Time	2.07	1	2.07	4.00	0.0593
A^2^	22.90	1	22.90	44.26	<0.0001
B^2^	13.12	1	13.12	25.35	<0.0001
C^2^	2.91	1	2.91	5.63	0.0278

**Figure 2:**
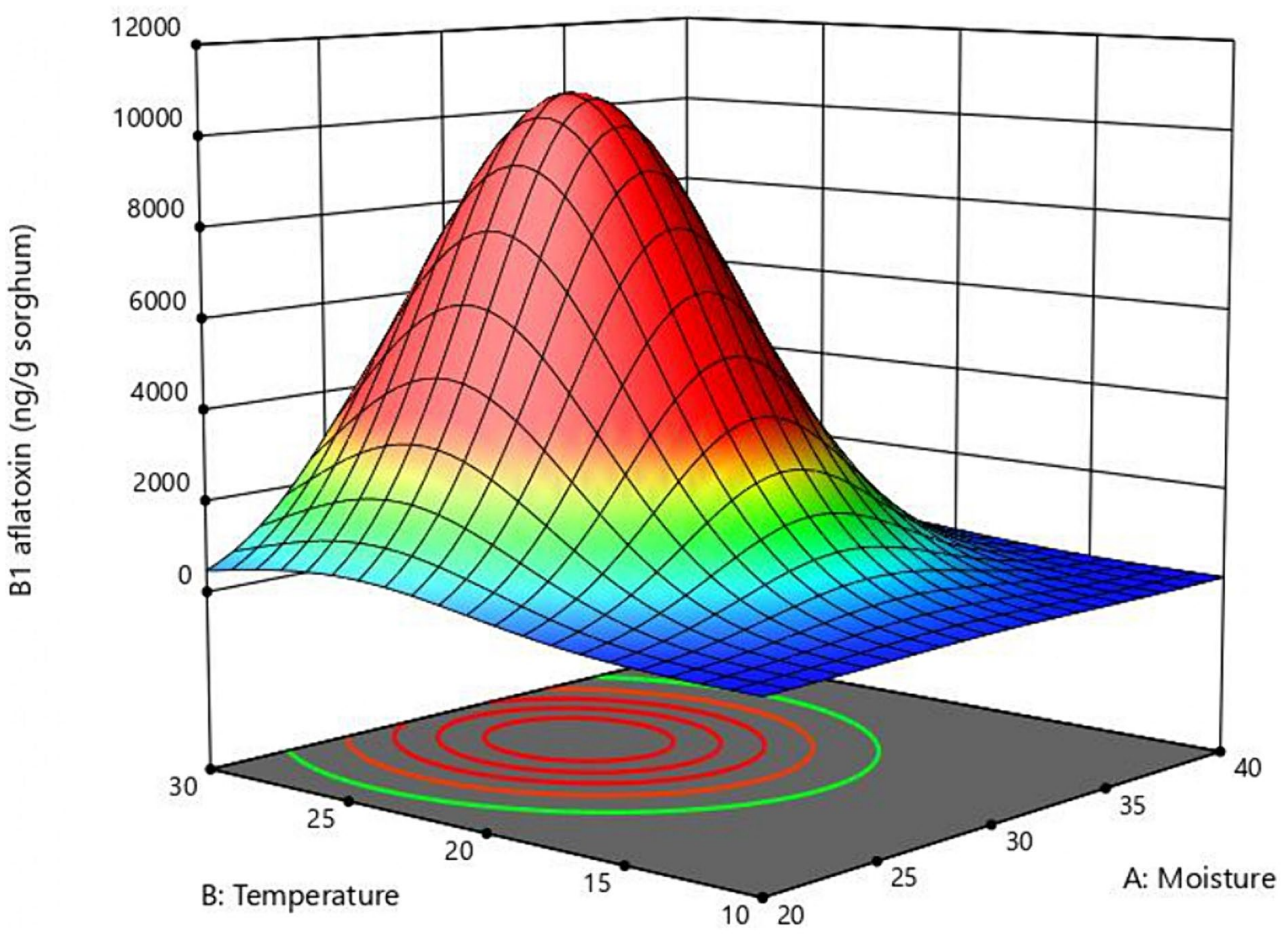
Response surface plot of the combined effect of moisture and temperature B1 aflatoxin production obtained with the central composite design.

The response surface curve shows the effect of two significant variables, moisture, and temperature, on aflatoxin B1 production (Figure 2) while the other two variables (inoculum and time) were held at 10^4^ conidia/g sorghum and 6 days, respectively. Values of moisture and temperature that allow optimal response in the assayed range are shown in red. The model predicted that the optimal values for aflatoxin B1 production in the assay range were moisture = 30%, temperature = 25°C, and time = 6 days. To validate the model B1 aflatoxin was quantified in mini-silos with 30% humidity incubated at 25°C for 6 days. Aflatoxin B1 obtained in these conditions was 10,643 ± 1,131 ng/g sorghum, which falls within the confidence interval and differs only 3.4% from the value predicted by the model.

**Figure 3:**
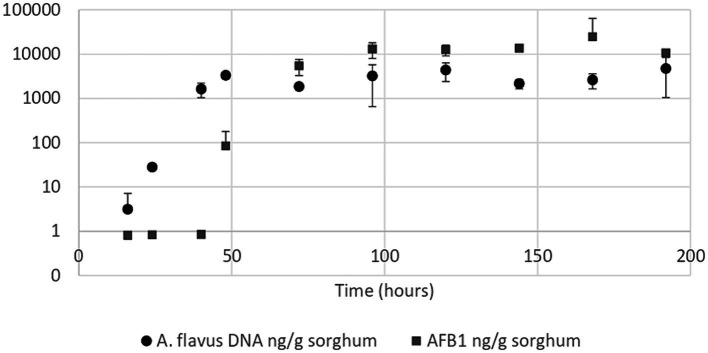
*Aspergillus flavus* growth curve (●) and aflatoxin B1 quantification curve (■) in high moisture sorghum mini-silos with 30% humidity incubated at 25°C. Curves were performed in duplicate. Bars show 95% confidence intervals.

*Aspergillus flavus* growth curve in sorghum mini-silos conducted under optimal conditions for aflatoxin B1 production (30% humidity and 25°C) revealed that the exponential phase began 24 h after the start of incubation and reached the stationary phase at 48 h (Figure 3). Aflatoxin B1 produced by the mold throughout its growth in these conditions was also determined. This metabolite began to be detectable at the onset of the stationary phase (48 h) at a concentration of 83.55 ng/g sorghum (Figure 3).

## 4 Discussion

In this work, *A. flavus* growth in sorghum mini-silos with air leakage was modeled at different temperatures. Among the models considered in this study, the Gompertz model was the most suitable. The Gompertz model (Winsor, 1932) has the advantage of presenting parameters with clear biological meaning, such as lag phase, growth rate, and maximum increase in microbial population. It is widely used to model bacterial and fungal growth (Arroyo et al., 2005; Ochoa-Velasco et al., 2018). For instance, it has been used to model fungal growth in stored barley grains at different temperatures and water activities (Wawrzyniak, 2021) and to study the growth of *Fusarium verticilloides* and *Rhizopus stolonifer* in the presence of antifungals such as thymol and carvacrol (Ochoa-Velasco et al., 2018). Moreover, Marín et al. (2006) modeled the growth of *Aspergillus carbonarius* and the production of ochratoxin A as a function of temperature in a synthetic grape medium using the Gompertz equation.

In the present study, the growth patterns fitted by the Gompertz equation showed that the maximum growth rate should be between 25 and 30°C. Below 25°C growth rates decreased significantly, reaching half of the maximum value at 20°C and a value 5 times lower at 15°C. These results suggest that the biomass of *A. flavus* at the end of the aerobic phase would be lower if this stage occurred at temperatures below 25°C. On the other hand, at 30°C, the growth rate was very similar to that obtained at 25°C, and no significant difference was observed. These results suggest that if the grain is ensiled under recommended moisture conditions for this purpose (28–32% moisture), and the aerobic phase takes place at temperatures between 25 and 30°C, the growth of *A. flavus* is likely to occur and aflatoxin B1 will be produced before the acidification phase begins.

Our work concluded that ensiled sorghum grains are a suitable substrate for *A. flavus* growth and aflatoxin production at least during the aerobic phase of the process. Contamination levels up to 30 ng/g of aflatoxin B1 were reported by Keller et al. (2012) when analyzing ensiled forage sorghum in São Paulo State, Brazil. High levels of aflatoxin B1 in silos are a potential hazard to animal health, however, studies of conditions that contribute to aflatoxin production in silos are scarce. Once aflatoxins are formed, they will not be eliminated in the silage. Therefore, designing strategies to inhibit the growth of aflatoxin-producing fungi is essential to reduce associated risks (Cheli et al., 2013).

The permitted limits of aflatoxin B1 in silos are governed by the limits provided for animal feed. The European Union and GMP (GMP, 2019) establish a limit of 5 ppb (ng/g) of aflatoxin B1 in feed for dairy cows, while for feed intended for other types of livestock, the limit is 20 ppb. In Uruguay, the Dirección General de Servicios Agrícolas del Ministerio de Ganadería, Agricultura y Pesca recommends a maximum limit of 20 μg/kg (ppb) of aflatoxins in feed for these animals in production, coinciding with the limits set by the FDA and USDA (FAO, 2003; Capelli et al., 2019). The method developed in this study for the quantification of aflatoxin B1 in sorghum allowed a quantification limit of (0.78 ± 0.05) ng/g, making it suitable for determining whether the samples comply with the stipulated regulations. Keller et al. (2012) found aflatoxin contamination in forage sorghum close to 1 ppb before ensiling. Given these findings, it is very important to have a sensitive technique that can detect this type of contamination in the starting material, as it alerts for possible risks in the final ensiled product.

In this work, artificially contaminated grains with *A. flavus* ensiled in different conditions, showed that, within the studied ranges, moisture, temperature, and the duration of the aerobic phase had a significant effect on aflatoxin B1 production. It was also found that the initial concentration of *A. flavus* spores in the material to be ensiled in the studied concentration range (10^2.32 spores/g to 10^5.68 spores/g) had no significant effect on the final concentration of aflatoxin B1. These results corroborate previous reports suggesting that very low levels of contamination with *A. flavus* in the material to be ensiled, even undetectable by plate count, could evolve to result in a high count of this fungus and detectable levels of aflatoxin B1, both in sorghum and maize (González Pereyra et al., 2008; Keller et al., 2013; del Palacio and Pan, 2020). These studies also describe *A. flavus* as the main fungal contaminant of ensiled material at the time of silo opening, demonstrating the difficulty of obtaining silage grains free of this fungus. The aforementioned highlights the importance of studying the effect of different variables on the development of *A. flavus* and aflatoxin contamination in sorghum silos.

In this context, Cheli et al. (2013) emphasize the need to develop predictive models regarding the presence of mycotoxins in silage based on storage conditions. The available data on predictive models of *A. flavus* growth and aflatoxin B1 production are mostly based on fungal growth and production data in plate agar culture media (Garcia et al., 2013; Lee et al., 2014). The data obtained under these conditions differ from what occurs in different matrices and storage systems, so it is necessary to validate or develop models directly in the study systems (Giorni et al., 2008; Aldars-García et al., 2016).

Until now, there has been insufficient data to enable the prediction of aflatoxin B1 production during the aerobic phase in high-moisture sorghum grain silos. In this work, as an approximation to the system under study, the model of high-moisture sorghum grain mini-silos was used, following the laboratory-scale mini-silo developed and validated by Petersson and Schnurer (1995) in their studies on biological control.

The results of the present work showed that aflatoxin production in the silages is maximum at 30% moisture, 25°C temperature, after 6 days in the aerobic phase. The optimal external temperature for toxin production determined in this study coincides with what is published in the Scientific Report of the European Food Safety Authority (EFSA) (Battilani et al., 2012), which mentions that most *A. flavus* strains produce the highest amount of aflatoxin at 25°C and that at 15°C and 30°C, the amount of aflatoxin B1 produced decreases.

The optimal moisture values for aflatoxin production in grains depend on the matrix under study, however, studies are scarce (Battilani et al., 2012). The optimal value for aflatoxin B1 production determined by the model developed in this work (30%) falls within the recommended moisture range for ensilage (28–32%) (Rovira and Velazco, 2012; Chalkling, 2016). As for the duration of the aerobic phase, it should be considered that in well-constructed silos, this phase generally lasts no more than 72 h after closure (Piltz and Kaiser, 2004). Therefore, in such silos, the predicted maximum production levels would not be reached. However, the growth curves of *A. flavus* and aflatoxin B1 production developed in this study demonstrated that at 48 h of incubation under optimal temperature and humidity conditions, the concentration of aflatoxin B1 in mini-silos artificially contaminated with *A. flavus* greatly exceeded the limits allowed by national legislation. These results are aligned with the data acquired from modeling *A. flavus* growth. The highest growth rate was obtained at 25°C, coinciding with the temperature of maximum aflatoxin production in the system. At this temperature, *A. flavus* reached the stationary phase at 48 h, at which point the concentration of aflatoxin B1 reached values close to 100 ppb. These results highlight the risk of the presence of this fungus in the material to be ensiled and emphasize the need to control its development in sorghum silos.

The growth curves of *A. flavus* in the mini-silos obtained in this study showed that the proliferation of the fungus is limited in the system, reaching maximum values equivalent to 10^7^ spores/g. Since the studies were conducted with pure cultures and there is no influence of metabolites produced by other microorganisms, it is postulated that the growth limitation is due to the depletion of essential nutrients for the fungus development. Although the entry of oxygen is not limited in the laboratory-scale mini-silo, as the fungus develops, the diffusion of gases through the material becomes difficult, which could also imply a growth limitation due to low availability of oxygen.

The final fungal contamination values obtained in commercial silos are normally lower than this value since there is competition for oxygen or nutrients with other microorganisms present, which can also produce metabolites that limit fungal growth. However, fungal contamination values exceeding 10^6 propagules/g after fermentation have been detected in high-moisture sorghum and maize grain silos (del Palacio et al., 2016; Ferrero et al., 2019). These values exceed the maximum permissible limit for fungal counts in good practices for animal feed handling (GMP, 2019), emphasizing the need for a strategy to reduce fungal growth in these systems.

In summary, the findings from this study highlight the capacity of *A. flavus* to grow in high-moisture sorghum silage with air leakage, resulting in the production of aflatoxin B1. Notably, the optimal conditions for aflatoxin production were observed at 25°C and 30% moisture, which coincides with the recommended humidity for constructing high-moisture silos. Furthermore, the results revealed that *A. flavus* reached the stationary phase at 48 h under these conditions, resulting in an aflatoxin B1 concentration that exceeded by nearly fivefold the permissible limit set by regulations. Given that the aerobic phase typically persists for a maximum of 72 h following closure (Piltz and Kaiser, 2004), the utmost significance lies in maintaining good agricultural practices to ensure the initial material’s hygiene quality and the incorporation of an inoculant to stimulate fermentation. This step is crucial for expediting oxygen reduction, thereby preventing *A. flavus* growth and the ensuing aflatoxin synthesis within the ensiled material.

## Data availability statement

The original contributions presented in the study are included in the article/Supplementary material, further inquiries can be directed to the corresponding author.

## Author contributions

MG: Conceptualization, Data curation, Formal Analysis, Funding acquisition, Investigation, Methodology, Software, Visualization, Writing – original draft, Writing – review & editing. CR: Conceptualization, Formal Analysis, Methodology, Software, Supervision, Writing – review & editing. JG-A: Conceptualization, Formal Analysis, Methodology, Resources, Software, Supervision, Validation, Writing – review & editing. SV: Conceptualization, Data curation, Formal Analysis, Funding acquisition, Investigation, Methodology, Project administration, Resources, Software, Supervision, Validation, Writing – original draft, Writing – review & editing.
