# Supplementary material for: Mitigating aflatoxin B1 in high-moisture sorghum silage: Aspergillus flavus growth and aflatoxin B1 prediction
Source: Front Microbiol. 2024 May 23;15:1360343. doi: 10.3389/fmicb.2024.1360343 (PMC11153755; doi:10.3389/fmicb.2024.1360343)
Supplement: Supplementary file 1 [file Table_1.DOCX]

Supplementary Material

Mitigating aflatoxin B1 in high-moisture sorghum silage: *Aspergillus flavus* growth and aflatoxin B1 prediction

Mariana Gonda^1^, Caterina Rufo^2^, Jose L. Gonzalez-Andujar^3^, Silvana Vero^1*^

*** Correspondence:**Corresponding Author
svero@fq.edu.uy

# Supplementary Tables

Table S1. Estimated values for parameters of the Gompertz model at different growth temperatures. A, upper asymptote, log_10_ (DNA/g sorghum); µ máx, maximum growth rate, 1/h; λ,lag phase, h.

| Temperature | Parameters | Standard error |
| --- | --- | --- |
| 15°C | A = 3.48 ** | 0.85 |
|  | µ máx = 0.03 *** | 0.003 |
|  | ʎ = 63.77 *** | 3.93 |
| 20°C | A = 3.90 *** | 0.10 |
|  | µ máx = 0.085 *** | 0.006 |
|  | ʎ = 17.90 *** | 1.24 |
| 25°C | A = 3.48 *** | 0.057 |
|  | µ máx = 0.17 *** | 0.025 |
|  | ʎ = 15.37 *** | 1.29 |
| 30°C | A = 4.52 *** | 0.14 |
|  | µ máx = 0.13 *** | 0.01 |
|  | ʎ = 8.01 *** | 0.67 |

(***) p-value < 0.001, (**) p-value < 0.01

Table S2. Central composite design matrix, B1 aflatoxin production obtained.

| Trial | Humidity (%) | Temperature (°C) | Time (days) | Inoculum 10^x^ (conidias/g) | B1 Aflatoxin (ng/g) |
| --- | --- | --- | --- | --- | --- |
| 1 | 40.0 | 10.0 | 8.0 | 3.0 | 0.4 |
| 2 | 40.0 | 10.0 | 4.0 | 3.0 | 0.1 |
| 3 | 40.0 | 10.0 | 4.0 | 5.0 | 0.4 |
| 4 | 30.0 | 36.8 | 6.0 | 4.0 | 32.7 |
| 5 | 46.8 | 20.0 | 6.0 | 4.0 | 0.6 |
| 6 | 40.0 | 30.0 | 4.0 | 5.0 | 0.5 |
| 7 | 30.0 | 20.0 | 9.4 | 4.0 | 496.8 |
| 8 | 30.0 | 20.0 | 6.0 | 4.0 | 1894.7 |
| 9 | 20.0 | 30.0 | 4.0 | 5.0 | 323.1 |
| 10 | 30.0 | 20.0 | 2.6 | 4.0 | 9.5 |
| 11 | 20.0 | 10.0 | 4.0 | 5.0 | 0.3 |
| 12 | 13.2 | 20.0 | 6.0 | 4.0 | 0.2 |
| 13 | 20.0 | 10.0 | 8.0 | 5.0 | 0.4 |
| 14 | 40.0 | 30.0 | 4.0 | 3.0 | 261.5 |
| 15 | 20.0 | 10.0 | 8.0 | 3.0 | 1.1 |
| 16 | 40.0 | 30.0 | 8.0 | 3.0 | 4.9 |
| 17 | 20.0 | 30.0 | 8.0 | 3.0 | 690.1 |
| 18 | 30.0 | 20.0 | 6.0 | 5.7 | 632.2 |
| 19 | 20.0 | 30.0 | 4.0 | 3.0 | 1.9 |
| 20 | 20.0 | 30.0 | 8.0 | 5.0 | 301.0 |
| 21 | 20.0 | 10.0 | 4.0 | 3.0 | 0.2 |
| 22 | 40.0 | 10.0 | 8.0 | 5.0 | 0.4 |
| 23 | 30.0 | 3.2 | 6.0 | 4.0 | 0.2 |
| 24 | 30.0 | 20.0 | 6.0 | 4.0 | 3789.4 |
| 25 | 40.0 | 30.0 | 8.0 | 5.0 | 17.1 |
| 26 | 30.0 | 20.0 | 6.0 | 4.0 | 1499.0 |
| 27 | 30.0 | 20.0 | 6.0 | 2.3 | 1156.4 |
